# Role of WNT10A-Expressing Kidney Fibroblasts in Acute Interstitial Nephritis

**DOI:** 10.1371/journal.pone.0103240

**Published:** 2014-07-23

**Authors:** Akihiro Kuma, Sohsuke Yamada, Ke-Yong Wang, Noriaki Kitamura, Takahiro Yamaguchi, Yoshiko Iwai, Hiroto Izumi, Masahito Tamura, Yutaka Otsuji, Kimitoshi Kohno

**Affiliations:** 1 Department of Molecular Biology, School of Medicine, University of Occupational and Environmental Health, Kitakyushu, Japan; 2 Second Department of Internal Medicine, Cardiology and Nephrology, School of Medicine, University of Occupational and Environmental Health, Kitakyushu, Japan; 3 Department of Pathology and Cell Biology, School of Medicine, University of Occupational and Environmental Health, Kitakyushu, Japan; 4 Bio-information Research Center, University of Occupational and Environmental Health, Kitakyushu, Japan; 5 Department of Occupational Pneumology, Institute of Industrial Ecological Science, University of Occupational and Environmental Health, Kitakyushu, Japan; 6 The President Laboratory, University of Occupational and Environmental Health, Kitakyushu, Japan; Northwestern University, United States of America

## Abstract

WNT signaling mediates various physiological and pathological processes. We previously showed that WNT10A is a novel angio/stromagenic factor involved in such processes as tumor growth, wound healing and tissue fibrosis. In this study, we investigated the role of WNT10A in promoting the fibrosis that is central to the pathology of acute interstitial nephritis (AIN). We initially asked whether there is an association between kidney function (estimated glomerular filtration rate; eGFR) and WNT10A expression using kidney biopsies from 20 patients with AIN. Interestingly, patients with WNT10A expression had significantly lower eGFR than WNT10A-negative patients. However, changes in kidney function were not related to the level of expression of other WNT family members. Furthermore, there was positive correlation between WNT10A and α-SMA expression. We next investigated the involvement of WNT10A in kidney fibrosis processes using COS1 cells, a kidney fibroblast cell line. WNT10A overexpression increased the level of expression of fibronectin and peroxiredoxin 5. Furthermore, WNT10A overexpression renders cells resistant to apoptosis induced by hydrogen peroxide and high glucose. Collectively, WNT10A may induce kidney fibrosis and associate with kidney dysfunction in AIN.

## Introduction

Acute interstitial nephritis (AIN) is a general cause of acute kidney injury (AKI). Patients with AIN can either completely recover or progress to chronic kidney disease (CKD) or end-stage renal disease (ESRD) [1]. Although several antifibrotic strategies have been proposed, there are no effective treatments for kidney fibrosis. Kidney fibrosis is generally considered to result from tissue inflammation and the tissue repair/wound healing responses [2]. Wound healing is a complex process involving several signal transduction systems, and ultimately can result in scar formation. The first phase begins with tissue damage caused by anti-inflammatory cytokines [3]. The next phase is defined by deposition of granulation tissue and new extracellular matrix (ECM) proteins. Acute inflammatory reactions are considered to be a part of early wound healing and play a key role in triggering fibrosis. Since prevention of the initial fibrotic process in AIN may lead to retention of kidney function, we investigated what kinds of signaling systems mediate kidney fibrosis associated with AIN.

There are two major WNT signaling pathways, the canonical (involving β-catenin) [4] and the non-canonical pathways (independent of β-catenin) [5], [6]. WNT is a family of highly conserved glycoproteins, and 19 WNT members have been identified so far in humans [7]. The WNT/Frizzled signal transduction system is a highly complex cascade that is fundamental for a wide variety of physiological processes, as well as pathological states. Some WNT proteins were previously investigated in kidney interstitial fibrosis [8], [9], but the relation between WNT10A and kidney fibrosis has not been determined. We asked whether WNT10A promotes kidney fibrosis. We previously reported that WNT10A is expressed in dermal fibroblasts, and that α-SMA-positive cells are involved in wound healing [10]. Myofibroblasts, or activated fibroblasts with α-SMA expression, differentiate from diverse sources including local interstitial fibroblasts, vascular pericytes, and endothelial cells [11]. Furthermore, they produce ECM (collagen and fibronectin). Therefore, we hypothesized that WNT10A expression in myofibroblasts may also play an important role in tissue repair and the fibrotic processes associated with AIN.

Here, we discovered that WNT10A is expressed in kidney fibroblasts expressing α-SMA. Patients with WNT10A expression had a significantly lower estimated glomerular filtration rate (eGFR) than WNT10-negative patients. We investigated mechanism of fibroblasts (COS1) behavior with WNT10A expression.

## Materials and Methods

### Cell culture and conditioned media

COS1 cells (kidney fibroblasts of African green monkey) were purchased from ATCC (VA, USA) and cultured in Dulbecco’s modified Eagle’s medium (Nissui Seiyaku, Tokyo, Japan) containing 10% fetal bovine serum and 5.5 mM glucose. Low glucose medium (LM) contained 5.5 mM glucose, moderate-high glucose medium (MM) contained 11 mM glucose, and high glucose medium (HM) contained 22 mM glucose. Cell lines were maintained in a 5% CO_2_ atmosphere at 37°C.

### Plasmid construction and transfectants

The WNT10A cDNA expression plasmid was constructed by PCR as described previously [10]. The PCR product was cloned into the pGEM-T easy vector (Promega, WI, USA). The full-length cDNA fragment was recloned into the pEB-Multi vector (Wako). COS1 cells were transfected with either vector (control cells) or pEB-WNT10A (WNT10A-overexpressing cells; COS1-10A) using the X-tremeGENE 9 (Roche, Basel, Switzerland) and cultured with normal medium for three days. After that, medium was changed to 300 µg/ml hygromycin contining medium and cultured for 21 days. The resulting colonies were isolated and the cellular expression levels of WNT10A were confirmed by Western blotting with an anti-WNT10A antibody.

### Preparation of human kidney samples

Patients (Japanese) who were hospitalized with AKI in the Department of Nephrology of University of Occupational and Environmental Health (UOEH) hospital in Kitakyushu, Japan, received kidney biopsy to diagnose the cause of AKI between January 2007 and March 2013. Human kidney tissue samples were examined in the department of nephrology and pathology at UOEH. The diagnosis was re-evaluated and confirmed by at least three board-certified nephrologists and one board-certified surgical pathologist. All the intended procedures of the present study, including use of specimens from human subjects, were approved by the Ethics Committee of UOEH in Kitakyushu, Japan. Especially, written consent of next of kin for research use of the materials was obtained according to the guidelines of the Japanese Society of Pathology and also approved by the Ethics Committee of UOEH, where the biopsy was performed.

### Histology and Immunohistochemical staining

Deparaffinized and rehydrated 3-µm sequential sections from kidney samples were stained with hematoxylin and eosin (HE) or Masson’s trichrome stain, or underwent immunohistochemistry (IHC) preparations. IHC was performed as previously described [12]. For IHC performed by the antibody-linked dextran polymer method (EnVision; DAKO, Glostrup, Denmark), samples were incubated in 10% H_2_O_2_ for 5 min to block endogenous peroxidase activity. Then, these sections were rinsed and incubated with the indicated antibodies for 30 min, respectively. The second antibody-peroxidase-linked polymers were then applied, and the sections were incubated with a solution consisting of 20 mg of 3.3′-diaminobenzidine tetrahydrochloride, 65 mg of sodium azide, and 20 ml of 30% H_2_O_2_ in 100 ml of Tris-HCl (50 mM, pH 7.6). After counterstaining with Mayer’s haematoxylin, sections were observed under a light microscope. The immune-reactivity with WNT proteins and α-SMA in each case were assessed semi-quantitatively by evaluating the proportion of positive cells over total stromal cells. For WNT proteins or α-SMA expression, positive areas that were equal to or more than 10% or 50% were considered positively stained respectively, in accord with the performance of receiver operating characteristic (ROC) curve analyses. Finally, to assess the co-localization of both proteins in the same or at least in adjacent fibrogenic cells (i.e., myofibroblasts), we stained tissues in consecutive sections with rabbit polyclonal anti-WNT10A antibodies and mouse monoclonal anti-human α-SMA, respectively, as previously described [13]. For fibronectin expression, positive areas that were equal to or more 30% were considered positively stained. All histological and immunohistochemical staining was evaluated by two independent observers (a certified surgical pathologist and a nephrology doctor) using a blind protocol design (observers were blinded to the clinicopathological data).

### Immunofluorescence staining

Control and WNT10A-overexpressing cells was cultured on 6 well-plate for 72 hours and fixed with 15% formalin for 10 minutes after washing with PBS. Cells were incubated with 0.2% (v/v) Triton X-100 with PBS for 10 minutes at room temperature. Cells were treated with 4% (w/v) skimmed milk (DS Pharma Biomedical Co., Ltd., Osaka, Japan) in PBS and incubated for 30 minutes at room temperature, washed with PBS, and incubated for 1 hour at room temperature with each primary antibody (anti-WNT10A and anti-fibronectin antibody). Primary antibodies were used at a 1∶100 dilution. Cells were reacted with Alexa 488 conjugated anti-rabbit IgG for 45 minutes at room temperature. After washed with PBS containing DAPI, specimens were observed.

### Western blotting analysis

Whole-cell lysates were prepared as previously described [14]. The 50 µg of protein from whole-cell lysates were separated by sodium dodecyl sulfate-polyacrylamide gel electrophoresis and transferred to polyvinylidene difluoride microporous membranes (Millipore, MA, USA). The blotted membranes were treated with 5% (w/v) skimmed milk in 10 mM Tris, 150 mM NaCl and 0.2% (v/v) Tween 20, and incubated for 1 h at room temperature with the primary antibody. anti-β-actin (Santa Cruz, CA, USA) antibody was used as an internal control at a 1∶5,000 dilution. The membranes were then incubated for 45 minutes at room temperature with a peroxidase-conjugated secondary antibody, and visualized using an ECL kit (GE Healthcare Bio-Science, Buckinghamshire, UK). The detection was performed with an LAS-4000 mini (Fujifilm, Tokyo, Japan).

### Antibodies

For western blotting and IHC, the following antibodies were used: anti-WNT10A (SAB3500393) was purchased from Sigma-Aldrich (MO, USA), anti-WNT1 (PAB11850) and anti-WNT3 (PAB7032) were purchased from Abnova (Taipei, Taiwan), and anti-WNT4 (sc-13962), anti-fibronectin (sc-9068), anti-COL1A1 (sc-25974), anti-COL3A1 (sc-28888), anti-PRDX2 (sc-23967), anti-PRDX4 (sc-23974) and anti-caspase9 (sc-56076) were purchased from Santa Cruz, and anti-caspase-3 (9662), anti-cleaved caspase-3 (9664), and anti-PARP (9532) were purchased from Cell Signaling Technology (MA, USA), anti-αSMA (M851) was purchased from Dako, and anti-PRDX3 (ab16751) was purchased from Abcam (MA, USA). To generate anti-PRDX1 and anti-PRDX5 antibodies, the synthetic peptides PDVQKSKEYFSKQK and GLTCSLAPNIISQL were used as immunogens, respectively, and the antisera obtained from immunized rabbits were affinity purified using the synthetic peptide antigens [15].

### PRDX5 siRNA knockdown

Double-stranded RNA oligonucleotides 25 base pairs long were commercially generated (Life technologies, CA, USA): 5′-GGAUGGCAUAGUGAAGGCCCUGAAU-3′ and 5′-AUUCAGGGCCUUCACUAUGCCAUCC-3′ for PRDX5#1; 5′-CCUGAGACGCUCAGCGGGCUAUAUA-3′ and 5′-UAUAUAGCCCGCUGAGCGUCUCAGG-3′ for PRDX5#3. siRNA transfections were performed as previously described [16], [17]. A sample of 5 µL of Lipofectamine 2000 (Qiagen, Hilden, Germany) was diluted in 250 µL Opti-MEM medium (Invitrogen, CA, USA) and incubated for 5 minutes at room temperature. Next, 250 pmol of siRNA and Lipofectamine 2000, serving as the control siRNA, were gently mixed in 250 µL Opti-MEM and incubated for 20 minutes at room temperature. Oligomer-Lipofectamine complexes and aliquots of 2×10^5^ WNT10A-overexpressing cells in 500 µL culture medium were combined and incubated for 48 hr. Whole cell extracts (50 µg) were prepared from cells after siRNA transfection, and western blotting was performed.

### Cytotoxicity analysis

Cytotoxicity analysis (water-soluble tetrazolium salt: WST-8 assay) was performed as described previously [18], [19]. Briefly, 2.5×10^3^ cells were seeded into 96-well plates. After 24 hours, to induce oxidative stress, cells were incubated with the indicated concentrations of H_2_O_2_ in the serum-free medium for 4 hours. After 48 hours, surviving cells were analyzed using a Cell Counting Kit −8 (Dojindo, Kumamoto, Japan) for 2 hours at 37°C. The absorbance was then measured at 450 nm.

### Cell proliferation assay

Cell lines were seeded into 12-well plates (2×10^4^ cells per well). For the purposes of analysis, the 0 hour was taken to be 24 hours after seeding. The cells were harvested with trypsin and counted every 24 hours.

### Statistical analysis

Statistical analyses of the data were carried out using STATA SE12 (Stata Co., TX, USA). Comparison between the two groups was performed by Mann-Whitney U-test or Student’s t-test. The *X^2^* test was used for dichotomous variables. A p-value <0.05 was considered significant.

## Results

### Patients with WNT10A expression had a lower estimated eGFR

**Figure 1:**
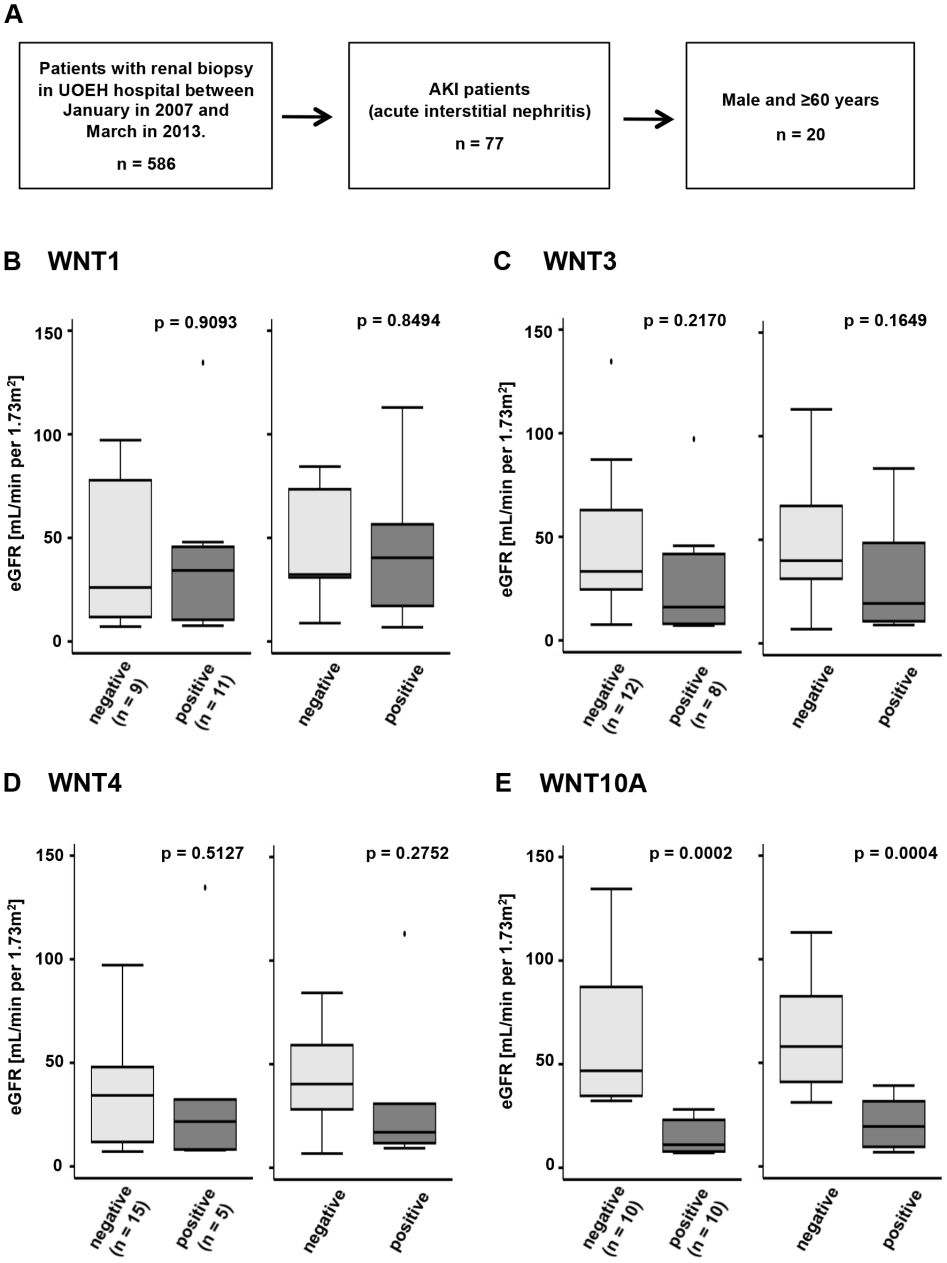
The relationship between WNT family gene expression and kidney function. (A) Patient selection method to evaluate eGFR among AIN patients. eGFR (mL/min per 1.73 m^2^) of AIN patients with (B) WNT1, (C) WNT3, (D) WNT4, and (E) WNT10A. (B) WNT1 negative-expression; n = 9, positive-expression; n = 11 (C) WNT3 negative-expression; n = 12, positive-expression; n = 8 (D) WNT4 negative-expression; n = 15, positive-expression; n = 5 (E) WNT10A negative expression; n = 10, positive-expression; n = 10. Light gray box indicates eGFR of patients without WNT protein expression, and dark gray box indicates eGFR of patients with WNT protein expression. The left panels show kidney function on kidney biopsy, and right panels show kidney function on discharge. Data of each category is shown in Table 2.

A total of 586 patients received kidney biopsy in the hospital of UOEH during a 75-month period starting in 2007. Of these patients, 77 were AKI and diagnosed with AIN. We investigated the relationship between eGFR and the expression of WNT proteins (WNT1, 3, 4, and 10A) in kidney tissue of 20 patients (Fig. 1A). We selected 20 male patients over 60 years of age because patient background was statistically matched. Primary causes of AIN were rapidly progressive glomerulonephritis (50%), drug-induced AIN (20%), and unknown AIN (30%) (Table 1). Interestingly, 10 patients with WNT10A expression had significantly lower eGFR (median; 11.12 mL/min per 1.73 m^2^, [interquartile range; 7.93–22.74 mL/min per 1.73 m^2^]) than that (46.81 [35.45–84.91]) of the 10 patients without WNT10A expression on kidney biopsy (Fig. 1E left panel). Concerning eGFR on discharge, patients with WNT10A expression also had significantly lower eGFR (19.27 [10.05–30.64] vs 57.87 [44.25–79.96]) (Fig. 1E right panel). However, expression of other WNT proteins (WNT1, 3, and 4) was not correlated with altered kidney function both on biopsy and discharge (Fig. 1B, C and D, Table 2). There was not statistical correlation between high WNT10A expression in kidney tissue and low eGFR (data not shown). Furthermore, three patients with WNT10A-positive tissue progressed to ESRD and were administered hemodialysis therapy (Table 1). No patient whose biopsied tissue was negative for WNT10A expression received renal replacement therapy. Thus, WNT10A expression may be correlated with poor prognosis of kidney function.

**Table 1:** Patient characteristics.

Characteristics	All	WNT10A(−)	WNT10A(+)	p-value
Number (n)	20	10	10	
Age (years, mean±S.D)	70.6±7.1	71.2±6.5	70.0±8.0	0.717
α-SMA positive (n, %)	10 (50)	1 (5)	9 (45)	0.0005
Fibronectin positive (n, %)	12 (60)	3 (15)	9 (45)	0.0062
Strongly interstitial fibrosis (n, %)	7 (35)	1 (5)	6 (30)	0.0191
Drug induced AIN (n, %)	4 (20)	1 (5)	3 (15)	0.264
Unknown AIN (n, %)	6 (30)	3 (15)	3 (15)	1.000
Crescentic nephritis (n, %)	9 (45)	5 (25)	4 (20)	0.653
MPA (n, %)	1 (5)	1 (5)	0 (0)	0.305
Administer maintenance HD (n, %)	3 (15)	0 (0)	3 (15)	0.060

AIN; acute interstitial nephritis, MPA; microscopic polyangiitis, HD; hemodialysis.

S.D; standard deviation.

**Table 2:** eGFR according to WNT proteins.

WNT protein	negative	positive	p-value
WNT1	26.06 [11.79–77.82]	34.28 [16.05–41.68]	0.9093
	32.31 [30.90–73.46]	40.40 [19.27–55.58]	0.8494
WNT3	33.29 [25.32–55.43]	16.05 [8.08–39.69]	0.2170
	39.91 [31.31–62.76]	19.27 [11.28–44.44]	0.1649
WNT4	34.28 [17.45–46.81]	21.66 [8.16–32.30]	0.5127
	40.40 [29.82–57.87]	17.14 [11.89–30.90]	0.2752
WNT10A	46.81 [35.45–84.91]	11.12 [7.93–22.74]	0.0002
	57.87 [44.25–79.96]	19.27 [10.05–30.64]	0.0004

eGFR; median [interquartile range] mL/min per 1.73 m^2^ above (on biopsy), below (on discharge).

### WNT10A and α-SMA expression in kidney fibroblasts associated with AIN

**Figure 2:**
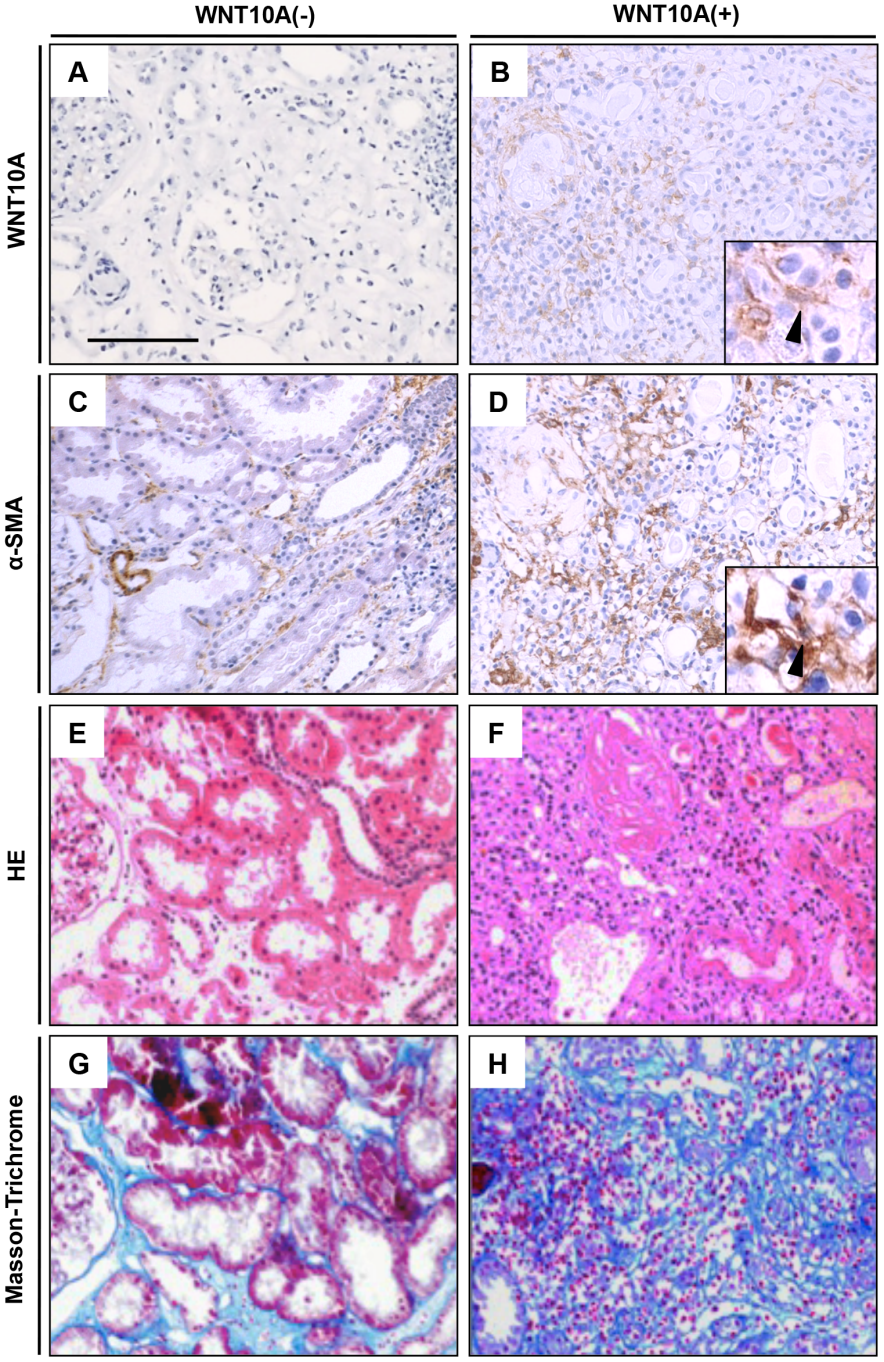
Histopathological analysis of kidney tissue from acute interstitial nephritis patients. Left panels are kidney interstitium without WNT10A expression, and right panels are kidney interstitium with WNT10A expression. (A–B); immunohistochemical staining for WNT10A, (C–D); immunohistochemical staining for α-SMA, (E–F); HE staining, (G–H); Masson-Trichrome staining. Arrow head is spindle cell with WNT10A and α-SMA positive respectively. All photos were taken at 200×. Scale bar is 100 µm.

WNT10A expression in kidney tissues obtained from AIN patient biopsies was much higher than that of samples from chronic kidney disease patients (Fig. 2B and Fig. S1). Inflammatory cells (primarily lymphocytes) were found associated with uriniferous tubules in tissues from patients with AIN (Fig. 2F). Fibroblasts that are α-SMA positive are thought to be myofibroblasts or activated fibroblasts that produce ECM components, such as type 1 collagen, type 3 collagen, and fibronectin. Some α-SMA-expressing cells were present at the interstitium of uriniferous tubules in kidneys without AIN (Fig. S2). Many more α-SMA-positive cells were identified in WNT10A-positive AIN cases (Fig. 2B and D) compared with WNT10A-negetive AIN cases (Fig. 2A and C). The consecutive IHC sections also demonstrated co-localization of both WNT10A and α-SMA in the same or at least in adjacent myofibroblasts/fibroblasts (Fig. 2B and D). Moreover, there was a significantly close positive correlation to the IHC WNT10A-positive and α-SMA-positive expression pattern (p = 0.0005) (Table 1). Finally, Masson-Trichrome staining showed that more ECM was found in WNT10A-positive AIN (Fig. 2H) than in WNT10A-negetive AIN (Fig. 2G). WNT10A expression in kidney tissue had significantly strong kidney fibrosis (fibrosis area is equal or more than 20%) compared with WNT10A-negative expression (p = 0.0191) (Table 1). However, there was not statistical correlation between high WNT10A expression (positive area is equal or more than 30%) and strong kidney fibrosis (p = 0.7782) (data not shown). Thus, WNT10A protein expression in α-SMA-expressing fibroblasts may contribute to the kidney interstitial fibrotic process characteristic of AIN.

### Fibroblasts with WNT10A expression produce fibronectin

**Figure 3:**
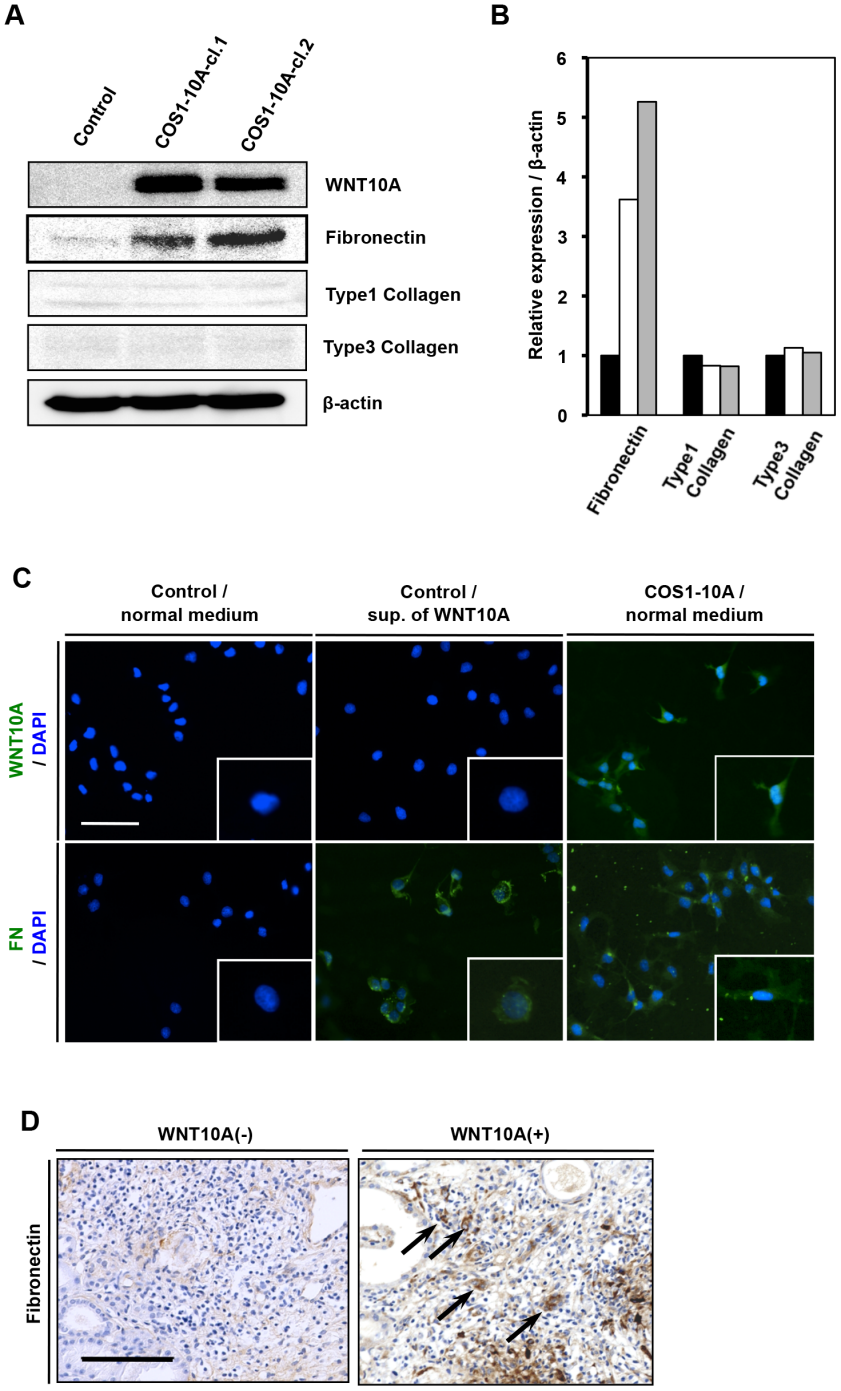
WNT10A overexpression enhances fibronectin expression in COS1 cells. (A) The expression of ECM components in WNT10A-overexpressing cells. Western analysis was performed using whole lysates obtained from control and WNT10A-overexpressing cells (COS1-10A). (B) Relative expression of fibronectin, type 1 collagen, and type 3 collagen, which were normalized to β-actin expression. The black bar shows control cells, white and gray bars showed COS1-10A-cl.1 and COS1-10A-cl.2 respectively. (C) Immunofluorescence staining for WNT10A (green), fibronectin (FN) (green), and DAPI (blue). Control cells treated with normal medium (left panel), control cells treated with supernatant (sup.) of WNT10A-overexpressing cells (middle panel), and COS1-10A cells treated with normal medium (right panel). Each cell was cultured for 72 hours. All photos were taken at 200×. Scale bar is 100 µm. (D) Immunohistochemical staining for fibronectin (brown) in AIN tissues. Kidney tissues from WNT10A-negative (left panel) or -positive (right panel) patients are shown. Arrows indicate fibronectin-positive cells. All photos were taken at 200×. Scale bar is 100 µm.

We investigated the involvement of WNT10A in the kidney fibrosis processes of AIN using COS1 cells, a cell line derived from kidney fibroblasts of the African green monkey. Two WNT10A-overexpressing COS1 cells (COS1-10A) were established (Fig. 3A). Interestingly, WNT10A overexpression enhances the expression of fibronectin (an ECM component) about three to five fold but does not alter the expression of type 1 and type 3 collagen (Fig. 3B). WNT10A is a secreted protein. In contrast to normal medium, supernatant from WNT10A-overexpressin COS1 cells induced fibronectin expression in untransfected COS1 cells (Fig. 3C). As expected, kidney tissues with WNT10A expression also expressed fibronectin (Fig. 3D). Furthermore, there was statistical positive correlation to the IHC WNT10A-positive and fibronectin-positive expression (p = 0.0062) in kidney tissues (Table 1). This indicates that WNT10A signaling plays a role in fibronectin synthesis and in activating stromagenic factors to induce fibrosis.

### WNT10A protects fibroblasts from oxidative stress

**Figure 4:**
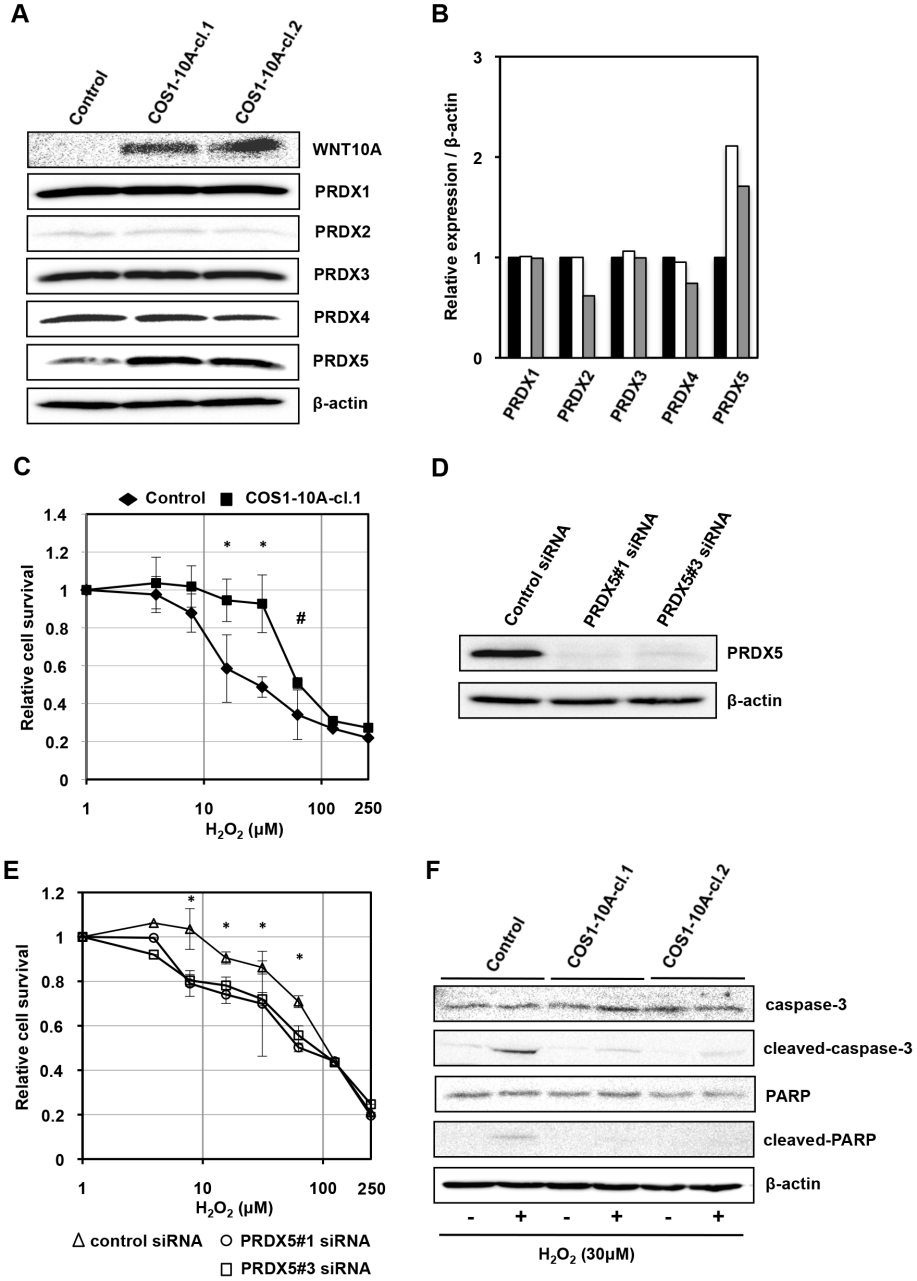
WNT10A protected fibroblasts against oxidative stress. (A) Western blot analysis of peroxiredoxin (PRDX)1, 2, 3, 4, and 5. Western blot analysis was performed using whole lysates obtained from COS1-vector (control) and COS1-10A cells. Only PRDX5 was increased with expression of WNT10A. (B) Relative expression of PRDX1, 2, 3, 4, and 5 normalized to β-actin expression. The black bar shows control cells, white and gray bars show COS1-10A-cl.1 and COS1-10A-cl.2 respectively. (C) The sensitivity of control and WNT10A-overexpressing cells (COS1-10A-cl.1) to H_2_O_2_. All values are the means of at least three independent experiments. Bars indicate standard deviation. *p<0.01 vs control. #p<0.05 vs control. (D) Downregulation of PRDX5 in WNT10A-overexpressing cells (COS1-10A-cl.1) by two kinds of siRNA. (E) The sensitivity of siRNA transfected cells to H_2_O_2_. All values are the means of at least three independent experiments. Bars indicate standard deviation. *p<0.05 vs PRDX5#1 and #3 siRNA. (F) Western blot analysis of caspase-3 and PARP expression. Western blot analysis was performed using whole lysates obtained from COS1-vector (control) and COS1-10A. Each cells were cultured in normal medium or medium containing hydrogen peroxide (30 µM) for 4 hours.

We previously reported that WNT10A expression is induced by treatment with hydrogen peroxide [10], suggesting that WNT10A signaling might induce the expression of antioxidant protein genes to protect cells. In this study, we found that WNT10A overexpression enhanced the expression of peroxiredoxin (PRDX) 5, but not PRDX 1, 2, 3 and 4 (Fig. 4A). COS1-10A cells expressed PRDX5 at approximately twice the level of control cells (Fig. 4B). PRDXs are a ubiquitous family of multifunctional antioxdant thioredoxin-dependent peroxidase that scavenge reactive oxygen species (ROS), suggesting that WNT10A overexpressing cells may be resistant against oxidative stress. As we expected, COS1-10A cells may be resistant to oxidative stress because WNT10A overexpression renders cells resistant to hydrogen peroxide (Fig. 4C). Down regulation of PRDX5 expression by siRNA sensitizes cells to hydrogen peroxide (Fig. 4D and E). COS1-10A cells expressed weakly active caspase-3 than control cells under hydrogen peroxide (Fig. 4F). WNT10A prevented apoptosis from oxidative stress via PRDX5 expression.

### WNT10A prevented apoptosis of fibroblasts from high-glucose stress

**Figure 5:**
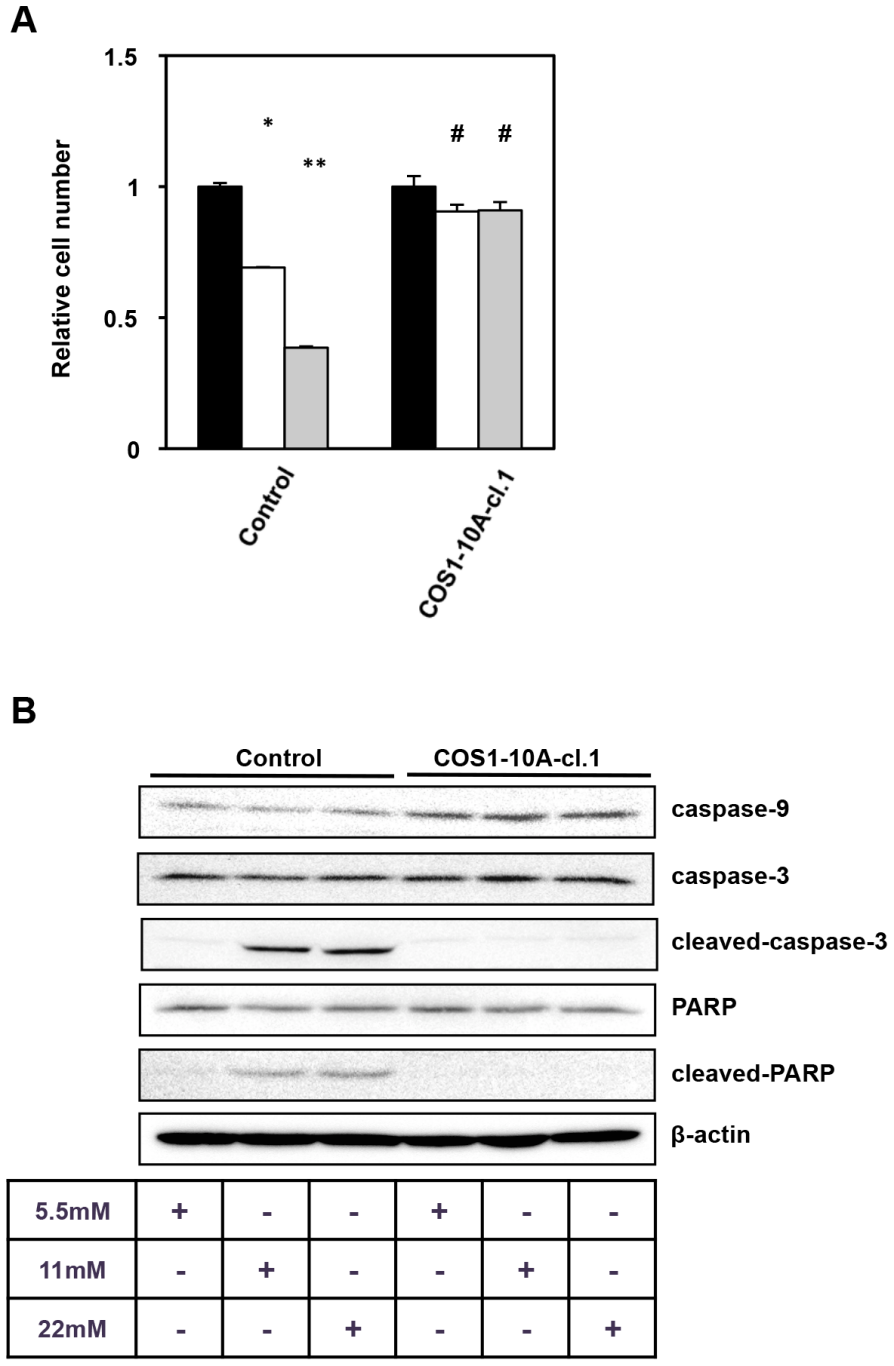
WNT10A protected fibroblasts against high glucose stress. (A) Proliferation assay. Control and WNT10A-overexpressing cells (COS1-10A-cl.1) (2×10^4^ cells/well) were seeded into a 12-well plate, and incubated in normal DMEM. After 24 hours, cells (0hr) were harvested, and other cells were cultured in each medium (5.5 mM, 11 mM, and 22 mM of glucose concentration) for 72 hours. Proliferative rates for 72 hours were compared. Black bar (5.5 mM), white bar (11 mM), gray bar (22 mM). All values are the means of at least three independent experiments. Bars indicate standard deviation. *p<0.001 vs 5.5 mM, **p<0.001 vs 5.5 mM and 11 mM, #p<0.05 vs 5.5 mM (B) Caspase and PARP expression. Whole lysates obtained from control and WNT10A-overexpressing cells treated with each medium (5.5 mM, 11 mM, and 22 mM of glucose concentration) were analyzed by western blotting.

High glucose triggers cell apoptosis, the generation of free radicals, and oxidative stress [20]. The control COS1 cells demonstrated poor survival following treatment with moderate- and high-glucose (MM and HM) compared with treatment with low-glucose medium (LM). However, COS1-10A cells survived even when treated with MM and HM, indicating that WNT10A overexpression protected fibroblasts from high glucose stress (Fig. 5A). Thus, high glucose stress leads to apoptosis of COS1 cells. As shown in Fig. 5B, control COS1 cells expressed caspase-3, cleaved caspase-3, PARP (poly ADP ribose polymerase), and cleavage-PARP compared with COS1-10A (Fig. 5B). WNT10A also prevented apoptosis from high glucose stress.

## Discussion

The kidney is exposed to several kinds of stresses including oxidative stress, high glucose stress, hypoxic stress, and uremic stress. Oxidative stress in the kidney is considered a major cause of kidney injury and inflammation [21]. The pathogenesis and recovery processes of AKI is regulated by a wide variety of pathways in various inflammatory cells that produce cytokines/chemokines and ROS. Tissue-specific fibrosis is differentially regulated by intrinsic and extrinsic mechanisms that involve organ-specific autocrine and paracrine systems. The molecular mechanisms that determine progressive CKD after AKI are largely unknown. A previous report showed that persistently high profibrotic signaling activity induces paracrine signaling that drives fibroblasts proliferation and inflammation [22]. WNT signaling is persistently active in areas exposed to stress. However, following AKI, WNT signaling becomes widely activated in kidney [23]. Since WNT10A induces angio/stromagenesis [10], we considered the possibility that there may be a relationship between WNT10A, tissue fibrosis, and inflammation. In contrast to AIN tissue, WNT10A hardly expressed in kidney of chronic kidney disease (Fig. S1). Therefore, we hypothesized that WNT10A-positive cells are found in proximity to areas of severe tissue injury, and that secreted WNT10A might be involved in fibrotic synthesis on AIN.

Tissue repair and fibrosis from injury involves multiple evolutionarily-conserved processes. Hydrogen peroxide can function as a chemoattractant following injury to transmit an immediate damage signal. After the initial response, angio/stromagenesis occurs leading to formation of new blood vessels, the appearance of (myo) fibroblasts, and the ECM gradually forming granulation tissue. The tissue remodeling after granulation is regulated by contractile fibroblasts called myofibroblasts [24]. The sources of renal myofibroblasts are local resident fibroblasts that arise from proliferation (50%), and nonproliferating myofibroblasts derived from bone marrow (35%) [25]. It has been proposed that the remaining progenitor population is derived from circulating fibrocytes, local pericytes, and resident epithelial cells, although this remains to be fully characterized [26], [27]. α-SMA expression induces fibroblasts into myofibroblasts [28]. Persistent myofibroblasts activation results in hypertrophic scars including keloid tissue or tissue fibrosis. Local activating fibroblasts express α-SMA and become fibrotic tissue [29]. Activation of WNT-β-catenin signaling has been reported in kidney and skin fibrosis [30], [31]. WNT10A expression in kidney tissue associated with α-SMA and fibronectin expression, and interstitial fibrosis (Table 1). The patients with WNT10A expression showed significantly lower eGFR than patients without WNT10A expression on kidney biopsy (Fig. 1 and 2), suggesting that WNT10A may induce persistent myofibroblasts activation leading to tissue fibrosis.

TGF-β and connective tissue growth factor are cytokines that up-regulate fibronectin expression during fibrogenesis [32]. As shown in Fig. 3, WNT10A overexpression enhances the expression of one of the ECM components, fibronectin. This data supports relation between WNT10A and fibronectin expression in AIN kidney tissue (Fig. 3D and Table 1). Consequently, WNT10A increases synthesis of interstitial fibrosis. Fibronectin has two different forms, cellular and plasma fibronectin. Cellular fibronectin is expressed by fibroblasts and is deposited locally into the ECM [33]. In general, locally-produced cellular fibronectin appears at the injury site within the early phase after tissue injury [34]. Plasma fibronectin is synthesized by hepatocytes and secreted into the blood plasma. Plasma fibronectin levels have been shown to increase after major trauma resulting in vascular tissue damage and inflammation such as atherosclerosis and ischemic diseases [35], [36]. Previous studies suggested that mechanical stress contributes to injury and fibrosis by inducing epithelial-mesenchymal transition (EMT) via a mechanism driven by TGF-β1 and WNT signaling [37], [38].

PRDXs are a ubiquitous family of multifunctional antioxidant thioredoxin-dependent peroxidases that scavenge reactive oxygen species (ROS). There are six distinct members located in various subcellular compartments. PRDX1, PRDX2, and PRDX6 are in the cytoplasm, and PRDX3 is in mitochondria. And PRDX4 is in endoplasmic reticulum and secreted. PRDX5 is expressed in mitochondria, the peroxisome, and the cytosol [39]. ROS are involved in initiation and implementation of apoptosis. PRDXs can efficiently eliminate hydrogen peroxide and participate in physiological processes such as apoptosis [40]. WNT10A promotes PRDX5 expression, and protects COS1 cells from oxidative stress (Fig. 4). Our study showed that WNT10A prevented COS1 cells from apoptosis by hydrogen peroxide. So, WNT10A may be helpful of fibroblasts to synthesize ECM under oxidative stress. As mentioned above, AKI is exposed several kinds of stresses including oxidative stress. WNT10A in kidney fibroblasts might play an important role in AKI/AIN.

We also investigated the relation between high glucose and WNT10A in COS1 cells. High glucose induces caspase-3-dependent apoptosis in fibroblasts, and activation of caspase caused by high glucose stress is independent of Fas/FasL signaling pathways system [41]. Activation of caspase-3 requires proteolytic cleavage of its inactive zymogen into active subunits. Cleaved caspase-3 causes apoptosis by degrading PARP. Our results show that WNT10A overexpression COS1 cells weaker expressed cleaved caspase-3 and PARP than control cells (Fig. 5B). WNT10A protected cells from apoptosis caused by high glucose stress. Consequently, a proliferation of WNT10A-overexpressing COS1 cells was small reduction than control cells (Fig. 5A).

3-hydroxy-3-methylglutaryl-coenzyme A (HMG-CoA) reductase inhibitors (statins) are used as therapy for hyperlipidemia. Statins inhibit kidney fibrosis and inflammation in rats [42], [43], and inhibits fibronectin expression in human airway fibroblasts [44]. Several studies have reported that statins are anti-inflammatory and block proliferation by G1 arrest of the cell cycle [45], [46], [47]. Our investigation also indicated that simvastatin suppressed proliferation of COS1 via inducing apoptosis regardless of WNT10A expression (data not shown). Our work suggests that it will be important to study the effects of statins during kidney fibrosis in a clinical setting.

In conclusion, we should point out that WNT10A expression leads to loss of kidney function due to excess tissue fibrosis. WNT10A enhances the synthesis of fibronectin. Furthermore, WNT10A expression in kidney is thought to promote kidney fibrosis following severe kidney dysfunction. COS1 cells with WNT10A expression are not induced to apoptosis against for hydrogen peroxide and high glucose. Thus, these findings might imply kidney fibroblasts with WNT10A-expression maintain their viability and cause kidney fibrosis under several kinds of stresses in AKI. We expect that WNT10A expression in kidney tissue may be a bio-maker of prognosis for AKI. Further studies to validate our conclusions in other model systems such as animal model and 3-D culture system will be critical for understanding the role of WNT10A in kidney fibrosis.

## Supporting Information

Figure S1 **Expression of WNT10A in the kidney tissues of a chronic kidney disease (CKD) patient (left panel) and AIN patient (right panel).** Immunohistochemical staining for WNT10A (brown). CKD patient had taken therapy for IgA nephropathy for a few decades. All photos were taken at 200×. Scale bar is 100 µm.
(TIF)

Figure S2 **Expression of α-SMA in the kidney tissues. Each tissue is minor glomerular abnormalities (left panel) and AIN with WNT10A expression (right panel). Few myofibroblasts with α-SMA expression can be seen in the circumference of uriniferous tubule in minor glomerular abnormalities.** Immunohistochemical staining for α-SMA (brown). All photos were taken at 200×. Scale bar is 100 µm.
(TIF)
