# Supplementary figures and images for: Role of WNT10A-Expressing Kidney Fibroblasts in Acute Interstitial Nephritis
Source: PLoS One. 2014 Jul 23;9(7):e103240. doi: 10.1371/journal.pone.0103240 (PMC4108433; doi:10.1371/journal.pone.0103240)

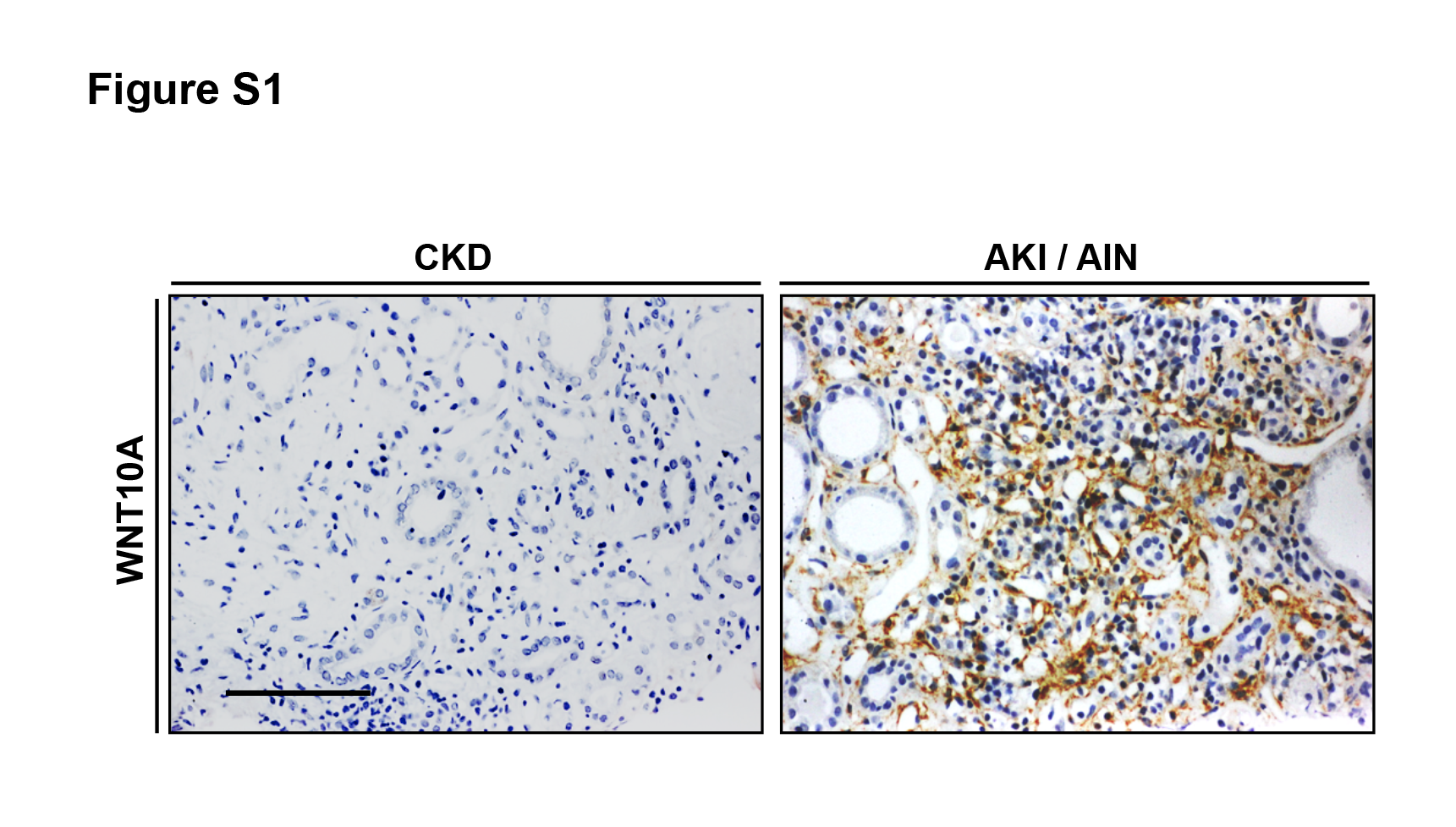

Supplement: Figure S1 — Expression of WNT10A in the kidney tissues of a chronic kidney disease (CKD) patient (left panel) and AIN patient (right panel). Immunohistochemical staining for WNT10A (brown). CKD patient had taken therapy for IgA nephropathy for a few decades. All photos were taken at 200×. Scale bar is 100 µm. (TIF) [file pone.0103240.s001.tif]

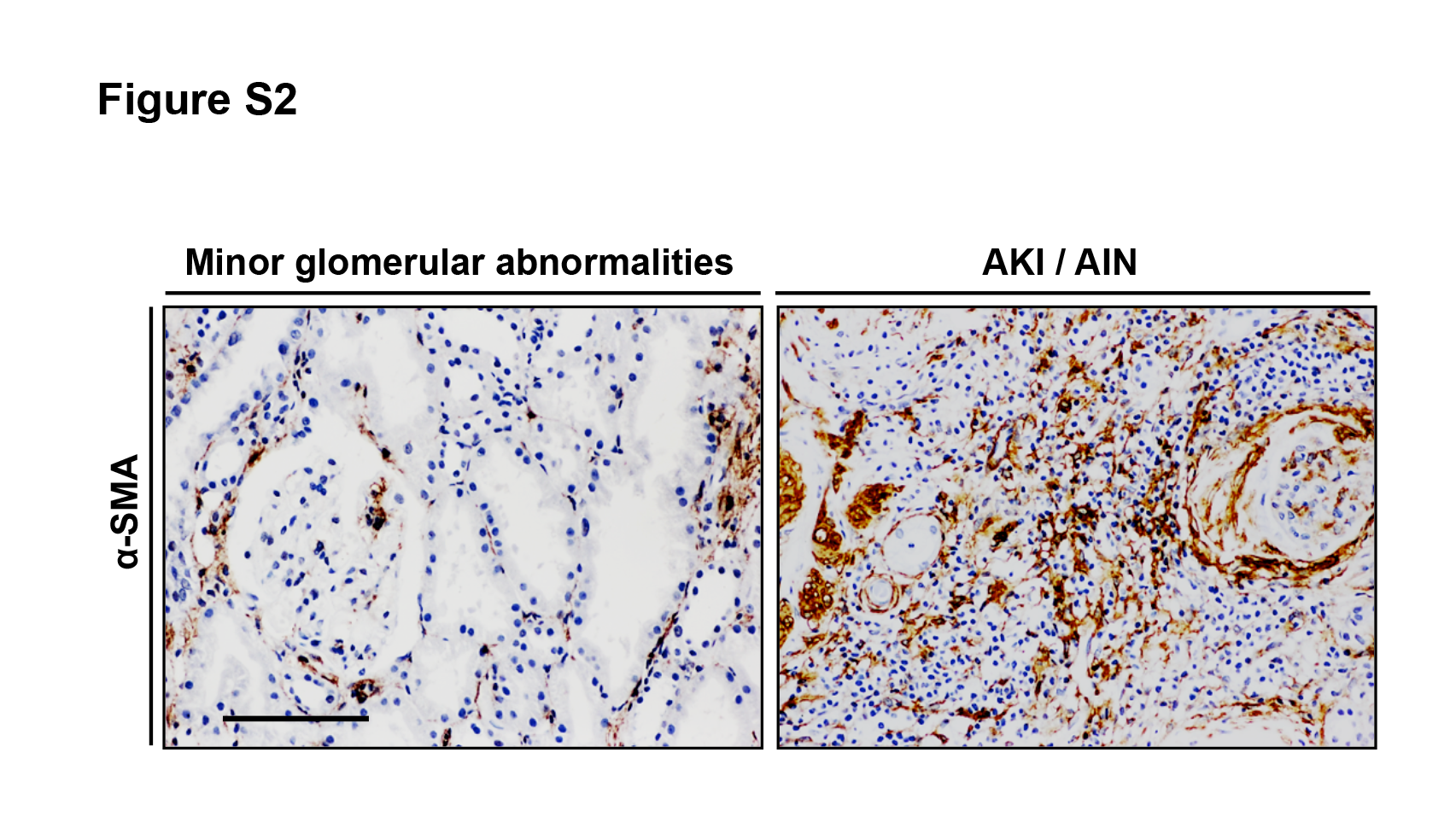

Supplement: Figure S2 — Expression of α-SMA in the kidney tissues. Each tissue is minor glomerular abnormalities (left panel) and AIN with WNT10A expression (right panel). Few myofibroblasts with α-SMA expression can be seen in the circumference of uriniferous tubule in minor glomerular abnormalities. Immunohistochemical staining for α-SMA (brown). All photos were taken at 200×. Scale bar is 100 µm. (TIF) [file pone.0103240.s002.tif]
